# Supplementary material for: Learning curve and surgical outcome of robotic assisted colorectal surgery with ERAS program
Source: Sci Rep. 2022 Nov 29;12:20566. doi: 10.1038/s41598-022-24665-w (PMC9709162; doi:10.1038/s41598-022-24665-w)
Supplement: Supplementary file 2 — Supplementary Information 2. [file 41598_2022_24665_MOESM2_ESM.zip › RAL Raw data-Ñ[▒K/079/HS_ñw╝╨░O▒Kñσ.pdf]

正式摘要(電子簽章：000000215970357)		
W52 / W52 ☐ 英文標題(注意:無英文姓名-請補病人基本檔!)	☐ 【讀取】憑證	

臺中榮民總醫院
出院病歷摘要

轉入號 : 轉出號 :

[redacted]

**(8)**流水編號	
**(9)**入院日期	110/04/28 (2021) 直腸外科 W52 - 121
**(10)**轉科日期	
**(11)**出院日期	110/05/04 (2021) 住院天數計 6 日

診斷

- 入院 :Colon Cancer, c T3 Nx M0, Stage IIB
- 出院 :Colon Cancer, c T3 Nx M0, Stage IIB, , s/p robotic assisted LAR on 2021/04/29

主訴

- occult blood positive in regular health examination

病史

- This 64 year-old male found herself occult blood positive in regular health examination. He went to our OPD for help. Abdominal CT showed tumor cannot be visulized. PET showed compatible with cancer involving the rectosigmoid junction. Under impression of malignant tumor of rectum, he was admitted to our ward for Robot Assisted Low Anterior Resection.

體檢發現

- Conjunctiva: mild injected, OU. SCH, os
  Cornea: clear. OU.
  Tear film: tear meniscus1mm with dry spots, OU.
  Schirmer's test: 2mmOD,2mmOS, in 5min.
  AC: deep/silent, OU.
  Pupil: RIC/LR+,OU.
  Lens: mild NS, ou
  Fundus: Pinkish disc, disc cupping: 0.3, OU.
  - General appearance :
    - easy looking , conscious clear , without cardiopulmonary distress
    - Vital signs自動帶入date 體溫(BT) 脈搏(P) 呼吸(R) 血壓(BP) 身高(BH) 體重(BW) BMI
      20210428 36.2℃ 61次/分 17次/分 122/84mmHg 179cm 92.9kg 29.0
    - Vital signs: B.P. - mmHg , P.R. 5 /min , R.R. 5 /min
  - HEENT :
    - Eyes: no anemic conjunctiva, no icteric sclera, normal EOM
    - ENT: no otorrhea, no rhinorrhea, no erythematous mucosa, no injected tonsil, no deformity
  - Neck: supple, freely movable, trachea midline,no deformity, no engorged jugular vein , no palpable thyroid or lymph node enlargement
  - Chest:
    - Inspection: Grossly normal, symmetric and fully expansion, no accessory muscle used, no operation scar, no spider angiomata, no gynecomastia
    - Palpation: no palpable mass or nodule, no subcutaneous emphysema, no tenderness
    - Ausculation: clear breathing sound
  - Heart:
    - Percussion: normal heart size
    - Ausculation: regular heart beat, normal S1, S2, no gallop, no murmur

-
  - Abdomen:
    - Inspection: in
      - flat contour, symetrically, no scar, no stria, no dilated veins, no rashes
    - Ausculation: normoactive bowel sound, no abdominal bruits
    - palpation: soft, no muscle guarding or rigidity, no local tenderness, no rebounding pain, no palpable mass, no palpable pulsation
    - Percussion: no shifting dulllness, no hepatomegaly, no splenomagaly
  -

Peripheral pulsation :	CA	BA	RA	FA	PA	PTA	DPA
Right side :	++	++	++	++	++	++	++/+++
Left side :	++	++	++	++	++	++	++/+++

  - Genito-anus: no tenderness or palpable mass lesion or rectal shelf, normal external genitalia, normal anal tone, brown stool

CRS CONDITION

-
  - Lung

手術日期及方法

- 手術日期及方法20210429 Robot Assisted Low Anterior Resection

住院治療經過

- After admission, pre-operation survey was done and revealed no contraindication of surgery. Therefore, Rectal Ca, s/p robotic assisted LAR was on 2021/04/29. After surgery, no complication noted, and wound pain was complained sometimes. Therefore, under stable condition, he was discharge and arranged OPD followed up.

合併症

一般檢查

- 累積報告 :

DATE	TIME	ALB	TP	BIL.T	BIL.D	ALKP	AST	ALT	LDH	CHOL	TG	HDL-C	LDL	UA	NH3	AMY
1100428	2118	4.5	6.9			98	17	28		186	44	62	110	6.6		
1100429	1916	3.7		0.8		74		19								

DATE	TIME	WBC	RBC	HGB	HCT	MCV	PLT	BLST	NEUT	BAND	SEG	LYM	MON	EOS	BAS
1100428	2117	6680	4.77	15.0	45.3				48.5			44.5	6.3	0.1	0.6
1100429	1916	12350		13.5			183		83.0			10.0	5.4	1.4	0.2

DATE	TIME	NA	K	CL	CA	BUN	CREAT	Mg	P	CRP	ESR	PCT	LIPASE	LACTATE
1100428	2118					14	0.96			0.121				
1100429	1916	142	3.6		8.4		0.92							

特殊檢查

病理報告

其它

出院時情況

- 3.改門診治療

出院指示

-

藥名	劑量	劑量單位	用法	頻次	數量	數量單位	現況	天數	單位
(口)Takepron OD tab 30mg	1	TAB	PO	QDAC	7	TAB	TH	7	天
Celebrex cap 200mg	1	CAP	PO	QD	7	CAP	TH	7	天
BethanechoL tab 25mg-JS	1	TAB	PO	TIDAC	21	TAB	TH	7	天
OCAS Harnalidge 0.4 mg	1	TAB	PO	QDAC	7	TAB	TH	7	天

出院計劃

主治醫師 2062F 蔣鋒帆 / 住院醫師 9760F 趙英如

正式摘要(電子簽章：000000215970357)

W52 / W52

doctorRank:B
